# Percent-Predicted 6-Minute Walk Distance in Duchenne Muscular Dystrophy to Account for Maturational Influences

**DOI:** 10.1371/currents.RRN1297

**Published:** 2012-02-02

**Authors:** Erik Henricson, Richard Abresch, Jay J. Han, Alina Nicorici, Erica Goude Keller, Gary Elfring, Allen Reha, Jay Barth, Craig M. McDonald

**Affiliations:** ^*^Department of Physical Medicine & Rehabilitation, University of California, Davis; ^‡^Associate Professor, Department of Physical Medicine and Rehabilitation, University of California Davis; ^§^Research Kinesiologist, Rehabilitation Research and Training Center in Neuromuscular Disease, Department of Physical Medicine & Rehabilitation, University of California, Davis; ^¶^Sr. Clinical Research Coordinator/Outreach Coordinator, Physical Medicine and Rehabilitation, University California of Davis; ^#^Executive Director, Biostatistics, PTC Therapeutics, Inc.; ^**^Director, Clinical Development, PTC Therapeutics, Inc.; ^††^Vice President, Clinical Development, PTC Therapeutics Inc. and ^‡‡^Professor and Chair, Department of Physical Medicine & Rehabilitation, University of California Davis Medical Center, 4860 Y Street, Suite 3850, Sacramento, CA 95817

## Abstract

We recently described a modified version of the 6-minute walk test (6MWT) for Duchenne muscular dystrophy (DMD) based partly on the American Thoracic Society (ATS) guidelines. This measure has shown reliability, validity and utility as a primary outcome measure in DMD clinical trials. Because loss of muscle function in DMD occurs against the background of normal childhood growth and development, younger children with DMD can show increase in distance walked during 6MWT over ~1 year despite progressive muscular impairment. In this study, we compare 6-minute walk distance (6MWD) data from DMD boys (n=17) and typically developing control subjects (n=22) to existing normative data from age- and sex-matched children and adolescents. An age- and height-based equation fitted to normative data by Geiger and colleagues was used to convert 6MWD to a percent-predicted (%-predicted) value in boys with DMD. Analysis of %-predicted 6MWD data represents a method to account for normal growth and development, and shows that gains in function at early ages represents stable rather than improving abilities in boys with DMD. Boys with DMD from 4-7 years of age maintain a stable 6MWD approximately 80% of that of typically developing peers, with the deficit progressing at a variable rate thereafter.

## **Background**

      A modified version of the American Thoracic Society (ATS) six-minute walk test (6MWT) has been effectively applied in Duchenne muscular dystrophy (DMD) clinical trials [1] [2] [3] .  An orientation video was employed to facilitate test performance in this population of children and adolescents with disease-related learning deficits.  Constant verbal encouragement was used to encourage subjects to walk as fast and as long as possible for 6 minutes.  A “safety chaser” was employed to assist the DMD subject up in the event of a fall.  Excellent feasibility, reproducibility, validity, and sensitivity were demonstrated [2] and longitudinal age-related changes in 6-minute walk distance (6MWD) over a 1-year period were described [3].  The measure has since been employed in international multicenter clinical trials and natural history studies in ambulatory boys with DMD [4] [5] [6] [7] [8] [9].  Mazzone et al have demonstrated that the North Star Ambulatory Assessment, a functional scale designed for ambulatory DMD boys, correlates better with 6MWD than with other commonly used timed motor performance evaluations (eg, 10-meter walk/run, supine to stand)[4] [8].  Collectively, these observations indicate that the 6MWT is becoming well-established as a clinically meaningful outcome measure in DMD. 

      One challenge in interpretation of longitudinal functional performance data in children and adolescents with DMD is that disease-related progressive loss of muscle function occurs against the background of normal growth and development.  Thus, patterns of change longitudinally for measures such as 6MWD can display a non-linear, inverted-“U” shape, where initial gains attributable to normal growth and development are eventually outpaced by losses due to disease progression.  Patients reach a functional “plateau” phase, which is followed by an overt and clinically appreciable decline in functional performance where changes in patients’ abilities are clearly distinct from changes in their healthy peers.  We previously showed 6MWD tends to improve up to age 7 and decline thereafter in boys with DMD [2] [3].  This tendency for 6MWD to increase prior to age 7 and to decrease in older patients was recently confirmed by Mazzone and colleagues [8].  Importantly, it is during this decline phase that treatment differences are most readily detectable in the context of interventional trials.

      Disease heterogeneity poses another challenge with regard to interpretation of functional performance data in DMD.  In particular, milder forms of the disease such as intermediate muscular dystrophy and Becker muscular dystrophy are associated with later manifestation and slower progression of symptoms [10].  In contrast, there are occasional steroid-naïve DMD patients that transition to the wheelchair as early as age 7 and begin their decline prior to age seven [11].  Heterogeneous steroid regiments may account for further increase in variability over time.  This variability in disease progression  may account for the increasing standard deviation for change in 6MWD observed over the course of a 12-month clinical trial [3].

      In pediatric specialties such as pulmonology [12] [13] [14], conversion of spirometry data (eg, forced expiratory volume in 1 second, forced vital capacity) to percent-predicted (%-predicted) values is commonly employed.  Here, this strategy is applied to help interpret 6MWD data in children and adolescents with DMD in the context of maturation.  As 98% of the variability in 6MWD is accounted for by stride length and cadence, which both have a direct relationship to disease progression in DMD [2] [15] [16], it was hypothesized that a %-predicted equation that considers both age and height (but not stride length) would be the most appropriate equation to apply to the DMD population.

## **Methods**

### *Evaluation of Participants*

      Participants were males between the ages of 4 to 12 years old at baseline, who either had DMD (documented by typical clinical presentation and either molecular genetic testing, muscle biopsy, or elevated serum creatine kinase and a family history of disease) or were non-diseased, typically developing controls.  Parents or guardians of all participants provided consent using a consent form approved by the UC Davis Institutional Review Board.  Participants underwent evaluation of anthropometrics and body composition prior to 6MWT.  6MWT followed the protocol previously described [2] [3].  Briefly, modifications of the ATS guidelines for the 6MWT included showing an introductory instructional video and adding a ‘safety’ chaser on the 25-meter course who walked behind the subjects and repeatedly reminded them to walk as fast as possible [1] [2] [3]. 

### *Identification of Normative 6MWD Data From the Literature*

      The literature was reviewed to identify studies of 6MWD in healthy children and adolescents that included 1) male children and adolescents in the same age range as the population we studied (ie, 4 to 12 years old); 2) a sample size sufficient to provide robust data across that age range; 3) a clear description of the level of effort elicited from subjects during the 6MWT (eg, comfortable self-selected pace, fast walk without running, walk or run); and 4) regression models explaining normative 6MWD across the age range based on age and height and not stride length which is strongly related to DMD-specific disease progression.  The goal was to have a prediction equation which would be influenced be growth but not disease progression over the course of a 12 month trial.  DMD subjects and control subjects tend to increase in height over 12 months.  DMD subjects show a decrease in stride length over 12 months while controls show an increase in stride length  over 12 months.  

### *Selection of a Suitable Normative Data Set*

      Percent-predicted normative 6MWD values were derived for typically developing control subjects based on the regression models identified above.  For any given 6MWT (ie, baseline or follow-up visit), %-predicted 6MWD was computed by dividing each subject’s actual 6MWD by the model-predicted normative 6MWD and multiplying by 100, using the model parameter values at that 6MWT.  Since the follow-up observation time was variable (median [range] = 66 [50–113] weeks for controls), linear interpolation or extrapolation was used to obtain values at exactly 52 weeks.  These %-predicted 6MWD measures were summarized (mean, SD, range, and mean sum of squares of deviations from 100%) to determine which regression model most closely fit our healthy control cohort 6MWD data and thus was suitable for application to our DMD cohort data.  

### *Evaluation of Patterns of %-Predicted 6MWD*

      Following selection of a suitable regression model, %-predicted 6MWD was computed for our DMD cohort.  Since the follow-up observation time was variable (median [range] = 58 [35–84] weeks for DMD), linear interpolation or extrapolation was used to obtain values at exactly 52 weeks.  Percent-predicted 6MWD versus age at baseline and the ~1-year follow-up visit was plotted to determine the age  at which boys with DMD show a substantial change in the rate of decline.

## **Results**

      Baseline and follow-up data at ~1 year were available from 17 participants with DMD and 22 healthy controls between 4 and 12 years of age.  Baseline mean (SD) 6MWD was substantially lower in boys with DMD than in healthy controls (352 [87] m vs 623 [66] m).  The mean (SD) change in 6MWD was -73 (135) m in boys with DMD vs 11 (33) m in healthy controls.  Participant characteristics and 6MWD results are summarized in Table 1.  

**Figure:**
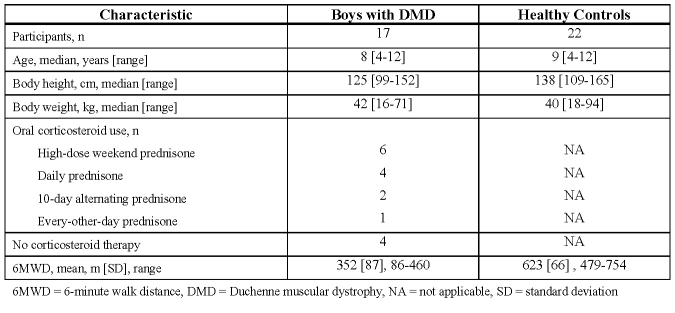
Table 1. Baseline Subject Characteristics

      The literature review identified 4 studies of 6MWD in healthy boys that provided reference equations [17].  These studies are summarized in Table 2.  Geiger et al evaluated 280 males between 3 to 18 years old and used a “far as possible” 6MWT instruction and constant feedback using a measuring wheel pushed by the participant [18].**  ** Li et al evaluated 805 males but did not study children <7 years old and used a “far as possible” 6MWT method with standard ATS feedback every 1 minute [19].  The studies by Priesnitz and by Ben Saad et al included smaller sample sizes, did not study children <6 years old, and used standard ATS “walk, don’t run” instructions and minute-by-minute feedback 6MWT methods [20] [21].  Of note, heart rate was included in the Li and Priesnitz regression models.  Applying these models to a population of boys with DMD could be problematic given the abnormal resting tachycardia and blunted response to exercise observed in DMD patients [2].  Thus, the study by Geiger et al was most similar to ours with regard to age range and 6MWT methods.  Also, Geiger et al studied subjects up to 18 years old, while the maximum age was 12 years old in the studies by Priesnitz and Ben Saad et al and 16 years old in the study by Li et al; although none of our subjects were >12 years old at baseline, the wider age range studied by Geiger may be advantageous with regard to calculation of %-predicted 6MWD in other studies in DMD. 

**Figure:**
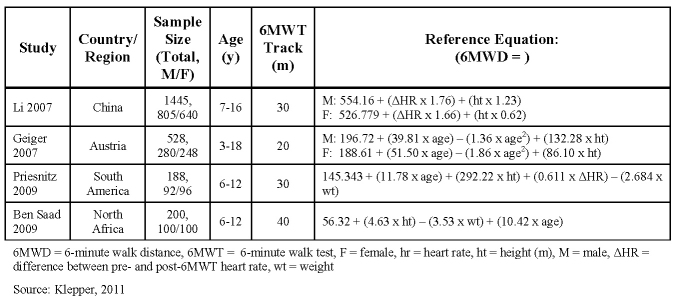
Table 2. 6MWT Methods and Reference Equations in Healthy, Typically Developing Pediatric Populations

      These reference equations were applied to the baseline 6MWD data from our healthy controls who were within the intersection of the age ranges in these 4 studies (ie, 7 to 12 years old).  The Geiger equation best predicted baseline 6MWD in our healthy controls (N=16), demonstrating a mean (SD) %-predicted 6MWD of 99 (7.9)% (Table 3).  By contrast, the Li et al and Priesnitz et al reference equations over- and under-estimated baseline 6MWD, respectively, for our healthy controls (N=16) (82 [6.2]% and 109 [6.4]%).  The Ben Saad et al reference equation slightly over-estimated baseline 6MWD for our healthy controls (N=16) (95 [5.9]%) but was comparable to the Geiger et al reference equation with regard to closeness of fit (ie, deviations from 100%).  Overall, these results indicated that the Geiger et al reference equation was the most appropriate for application to our 6MWD data.  Although the method by Ben Saad et al reference equation also fit our healthy control cohort data well, the different age range and 6MWT method used by Ben Saad et al favored selection of the Geiger et al reference equation for analysis of our DMD cohort data.

**Figure:**
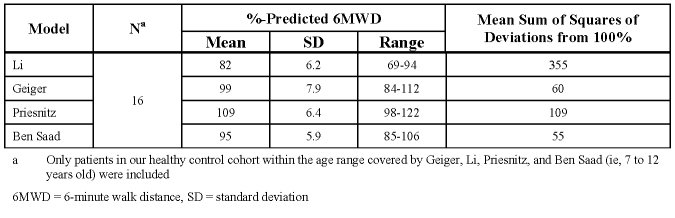
Table 3. %-Predicted 6MWD in the Study’s Healthy Control Cohort as Determined by Identified Regression Models

      Percent-predicted 6MWD scores for all observations in our DMD cohort were calculated using the Geiger method **(**Figure 1**)**.  Overall, mean (SD) %-predicted 6MWD was 61.2 (18.8)% at baseline and 47.3 (31.8)% at 1 year in patients with DMD.  The mean (SD) decline in %-predicted 6MWD over 1 year was 14 (21.0)% in this patient population.  By contrast, mean (SD) %-predicted 6MWD was 100 (7.5)% at baseline and 99 (8.0)% at 1 year in healthy controls (N=22).  The mean (SD) decline in %-predicted 6MWD over 1 year was 0.6 (4.5)% in this control population.

**Figure:**
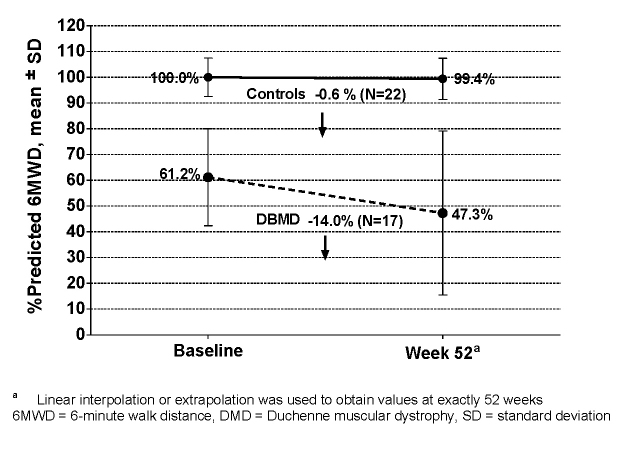
Figure 1. %-Predicted 6MWD in Boys with DMD and Healthy Controls at Baseline and Week 52

The reduction in ambulatory function for boys with DMD progresses with age as shown in Figure 2.  In this study, baseline %-predicted 6MWD ranged from 88% (460 m) in a 6-year old patient to 13% (86 m) in a 10-year old patient.  Absolute 6MWD increases in normally developing children as a function of growth across the entire age range (Figure 2a).  The figure also shows proportional one-year *increases* in 6MWD in boys with DMD to ~7 years of age, albeit at approximately 80% of control values.  Above age 7, most participants show 1-year * decreases* in 6MWD that are characteristic of DMD disease progression.  The graphical presentation of %-predicted 6MWD shows the “flattening” effect of adjusting for normal growth, thus changing the early performance increases in  absolute 6MWD to a pattern more reflective of “clinically stable” disease at ages less than ~7 years, followed by expected progressive declines (Figure 2b).

**Figure:**
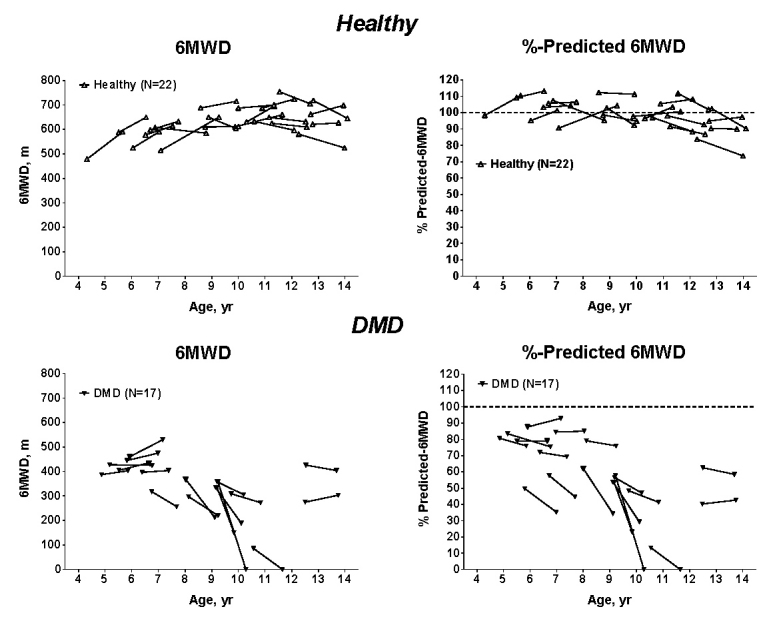
Figure 2. Changes in %-Predicted 6MWD in Boys with DMD from Baseline to Follow-Up Visit  

## **Discussion**

      Therapeutic intervention in patients with DMD during early childhood is likely to yield the most effective preservation of muscle.  Detection of treatment effects on muscle function at this stage of disease, however, is challenging.   Younger patients with DMD who are still growing are likely to show improvements over time in functional performance measures, such as 6MWD, until approximately 7 years of age regardless of treatment for DMD [3] [4] [8]. Meanwhile, older patients with DMD who are beyond the plateau phase may show overt decreases over time in functional performance measures, such as 6MWD.  Converting 6MWD to %-predicted 6MWD may help to distinguish normal growth and development from treatment effects.  Within the context of clinical trials, these age- and growth-related factors have the effect of increasing the variability of changes from baseline, thereby requiring larger sample sizes [22].   

       Furthermore, functional performance results have been used in the past to determine minimum threshold values that can predict future loss of function.  For example, a 10-meter run/walk time of ≥12 seconds has been correlated with an ~90% risk of losing ambulation during the following year [11].  This 12-second threshold, which theoretically translates to a 6MWD of 300 meters assuming the same pace can be maintained (ie, 12 seconds divided by 10 meters equals a velocity of 0.83 meters per second, which multiplied by 6 minutes equals 300 meters), has been used to justify exclusion criteria for clinical trials to help maximize the likelihood that participants will remain ambulatory during the course of a 1-year study.  Because it is likely the same pace cannot be maintained by a patient with DMD for 6 minutes, the 12-second threshold realistically translates to a 6MWD <300 meters.  Nonetheless, such thresholds based on absolute values ignore the fact that younger and older participants are on opposite sides of the “inverted U” distribution and therefore a 6MWD that reflects nearly normative function in the younger child may indicate a notable degree of  impairment in an older child.  Future studies should address whether minimum threshold values based on %-predicted values may be more appropriate for predicting loss of function in DMD. 

      Normal growth and development should be taken into account when interpreting absolute values of functional measures in patients with DMD, as the trajectory of changes in functional performance measures over 1 year may vary depending on age and related characteristics such as height.  The most straightforward way to account for normal growth and development is to evaluate the performance of a DMD boy relative to the typical performance of age- and height-matched healthy peers by calculating a %-predicted value.  While this is a common approach in pediatric specialties such as pulmonology [12] [13] [14], it has not yet been commonly employed in the functional assessment of individuals with neuromuscular diseases.

      When the ATS guidelines for conducting the 6MWT were published in 2002, it was not yet known whether change in 6MWD should optimally be expressed as an absolute value, a percentage change, or a change in the %-predicted value.  Citing a need for further research, the ATS guidelines recommended expressing change in 6MWD be expressed as an absolute value [1].  However, in the past few years, pediatric normative data have become available that may be used to calculate %- predicted 6MWD.  Among several regression models identified in the literature, the results presented here show that the Geiger equation is most suitable for use in calculating %-predicted 6MWD in boys with DMD.  Notably, the 6MWT method employed by Geiger encouraged subjects to walk as far as possible by providing  consistent feedback [18], a departure from the ATS guidelines and unlike the other normative studies but similar to the method for conducting the 6MWT in boys with DMD [2].  Perhaps due to this methodologic similarity, the Geiger equation very accurately predicted the 6MWD results in our healthy control cohort. 

      Although similar, the Geiger 6MWT method and ours are not identical.  For example, Geiger et al used a 20-m track, while our track was 25 m.  Geiger et al used encouragement in 1-minute intervals and a measuring wheel that displays the instantaneous walking distance, while our method employed a “safety chaser” who walked behind the participant and provided encouragement at 15-second intervals.  Despite these methodologic differences, based on our experience we recommend using the Geiger equation to determine %-predicted 6MWD in the DMD population, at least until a regression model based upon our 6MWT method has been developed; this work is ongoing.           

       Our previous reports of 6MWD data in boys with DMD at baseline and longitudinally over 12 months [2] [3], and 6MWD data subsequently reported by Mazzone et al [4], both indicated a moderate degree of increase in 6MWD over a 1-year period in younger children (ie, <7 years old) with DMD.  These data and normative data in typically developing children show that this increase is consistent with normal growth and development [3].  In the present study, we show that children with DMD as young as 4 years of age show on average a 20% deficit in 6MWD relative to healthy peers, and that this deficit remains somewhat stable until middle to late childhood.  Beginning between ages 7 and 8, however, the relative reduction in ambulatory function accelerates at an increasing pace to a point where adolescents between ages 10 and 12 may lose function relative to controls at 20% per year or more.  Understanding the  functional changes observed in DMD patients during their development is critical in clinical trial development, as the effect of therapeutic intervention in a given patient population can be more variable than anticipated (or more difficult to detect) depending on the maturational status of the DMD patient. 

      In comparison with our previous presentations of data from this cohort, we show that early gains in function that affect overall groupwise change estimates can be “flattened”, or normalized using %-predicted values.  While we obtained our data from a small and somewhat heterogeneous cohort, these results further demonstrate that increases in 6MWD are proportional to normal growth up to about age 7 in boys with DMD, which is consistent with the commonly held concept of the “honeymoon period” in DMD during which functional gains that result from growth and development keep pace with disease progression such that %-predicted 6MWD is stable at ~80% of healthy controls.  Past age 7, boys with DMD experience substantial declines in %-predicted 6MWD.  Additional longitudinal studies of %-predicted 6MWD using larger cohorts of boys with DMD across a wider age range are planned.     

## **Acknowledgements**

The authors thank the patients and volunteers who committed their time and effort, John Orr of Innovative Analytics, Inc. for his expertise in data management, and Peter Riebling of PTC Therapeutics for his manuscript support.

## **Funding**

This study was sponsored by PTC Therapeutics and funded in part by a grant from the Parent Project Muscular Dystrophy. Dr. McDonald (PI), Dr. Han, Mr. Henricson and Mr. Abresch were funded in part by grants from the U.S. Department of Education/NIDRR (#H133B031118, #H133B090001). 

## **Competing interests**

EK Henricson: Mr. Henricson has served as a consultant for Genzyme, Inc. and PTC Therapeutics, Inc.  RT Abresch: Mr. Abresch has served as a consultant for PTC Therapeutics, Inc.  J Han: Dr. Han has nothing to disclose.  A Nicorici: Ms. Nicorici has nothing to disclose.  EM Goude: Ms. Goude has nothing to disclose.  A Reha: Mr. Reha is an employee of PTC Therapeutics and holds financial interests in the company  GL Elfring: Mr. Elfring is an employee of PTC Therapeutics and holds financial interests in the company J Barth: Dr. Barth is an employee of PTC Therapeutics and holds financial interests in the company CM McDonald: Dr. McDonald has served on advisory committees for PTC Therapeutics, Inc., AVI BioPharma, Inc., GlaxoSmithKline, PLC., Prosensa, Shire HGT / Acceleron Pharma, Inc. and Halo Therapeutics  

## **Corresponding Author**

Erik Henricson, MPH  Department of Physical Medicine & Rehabilitation  University of California, Davis School of Medicine  Sacramento, California 95817  Telephone: 916-734-0384  Fax: 916-734-7838  E-mail: erik.henricson@ucdmc.ucdavis.edu

## **Key Words/Mesh Terms**

Height, Weight, Child (Preschool), Disease Progression, Follow-Up Studies, Gait/physiology*, Humans, Male, Muscular Dystrophy, Duchenne/physiopathology*, Walking/physiology* 

## **Abbreviations**

6MWT Six-minute walk test  6MWD Six-minute walk distance  ATS American Thoracic Society  DMD Duchenne muscular dystrophy  HR Heart rate  HT Height  SD Standard deviation  WT Weight 
